# An “Empress” of Rome

**Published:** 1904-08

**Authors:** 


An "Empress" of Rome.—A Kansas doctor quotes Shakespeare[?]. I extract the following, *verbatim,* from a pamphlet lately received, entitled "The Conjugal Relation, or Sexual Hygiene," by a Kansas doctor of some pretentions:

"We look with awe on other forms of perversion like that so graphically portrayed in the picture drawn by Shakespeare from life at the head of a Roman court, where the Empress—Anne Boleyn or Bullen, the second wife of Henry VIII—fair as a lily, in lewd embrace clasped to her bosom the murderous and licentious black man Aaron. This was in the far-off past, and yet, through the gloom of more than 2000 years, its horrid details are sufficient to fill us with loathing."

Oh! Oh! Oh! That is delicious! The unfortunate Anne Boleyn, the mother of England's most famous queen, referred to as *Empress of Rome* sixteen hundred years before she was born! Shades of Shakespeare, Gibbon and the Cæsars! but it is a good joke on you. In shade-land, I can imagine old Shake sulking and saying cuss words,—while Gibbon and the Cæsar boys are nudging him in the ribs and guying him. What do you suppose the learned writer was hitting at, anyway? Really, if I had a ten-year-old boy who didn't know better than that, I'd send him to Dr. Pilcher, in charge of the asylum for idiots at Winfield, Kans., to be bored for the simples.

In this same pamphlet I find several pages taken from my paper on "The Cause and Prevention of Rape; Sadism in the Negro," without acknowledgment, credit or even quotation mark. That is bad enough, but it is published (without date of publication) as a paper claimed to have been read several months prior to the publication of my paper. This indirectly asserts priority to the use of my own words, and is calculated and was intended to deceive. To be sure it is not true; but that seems to make no difference with the alleged author; he does not seem to mind a little thing like that. It is really not worth mentioning, and I would not mention it except to denounce it as an atrocious plagiarism. For, "Big fleas have little fleas on their backs to bite 'em," you know, as Saxe said, and we must be patient under that affliction of which, when told of a published criticism of him, the great John Hunter said: "Yes, we all of us have vermin that feed upon us."

